# An interpretable deep learning framework for multiclass bone marrow cytomorphology classification using EfficientNet and post hoc visualization techniques

**DOI:** 10.1016/j.jpi.2026.100710

**Published:** 2026-08-20

**Authors:** Saanie Sulley, Ghassan Tranesh

**Affiliations:** a National Healthy Start Association, Washington, DC, USA; b Health Information Management and Health Services Administration, CUNY School of Professional Studies, New York, NY, USA; c Integris Health Data Systems LLC, Chantilly, VA, USA; d Pathology and Laboratory Medicine, University of Arizona College of Medicine, Phoenix, AZ, USA

**Keywords:** Bone marrow cytomorphology, Hematopathology, Deep learning, Explainable artificial intelligence, Multiclass classification, Grad-CAM, SHAP, Cross-validation

## Abstract

**Background:**

Automated classification of hematopoietic cells presents unique challenges due to morphological overlap across maturation stages and interobserver variability. Whereas deep learning models have demonstrated promising performance in digital pathology, limited attention has been given to systematic interpretability analysis in hematological cytomorphology.

**Objective:**

To develop and rigorously evaluate an interpretability-centered deep learning framework for multiclass classification of 21 bone marrow cytomorphological cell types, incorporating cross-validation, formal statistical comparison of architectures, and multimodal visualization to assess both predictive performance and biological plausibility.

**Methods:**

We developed a multiclass deep learning framework using EfficientNet-B3 to classify 171,373 bone marrow single-cell images spanning 21 morphological categories. Model performance was evaluated using top-1 accuracy, top-5 accuracy, macro-F1, and weighted-F1 scores. To assess robustness, 5-fold stratified cross-validation was performed. EfficientNet-B3 was compared against ResNet50 and DenseNet121, with statistical significance assessed using McNemar's test and bootstrap confidence intervals. Interpretability was evaluated through Grad-CAM visualization of confusion pairs and SHAP-based feature attribution. Latent feature structure was examined using PCA, UMAP, and t-SNE projections.

**Results:**

On the held-out validation set, EfficientNet-B3 achieved top-1 accuracy of 87.6%, whereas cross-validated performance averaged 76.3% ± 0.27. Performance was statistically superior to both ResNet50 and DenseNet121, although the magnitude of improvement over DenseNet121 was modest. Most misclassifications occurred between morphologically adjacent classes, consistent with biological lineage continuity. Grad-CAM analysis demonstrated biologically plausible attention patterns in nuclear and cytoplasmic regions. Embedding projections revealed partial class clustering with expected overlap among transitional cell types.

**Conclusion:**

This study reframes deep learning-based hematopoietic classification as an interpretability-centered problem. By integrating cross-validation, statistical model comparison, and multimodal visualization, we provide a comprehensive framework for understanding both performance and failure modes in multiclass bone marrow cytomorphology classification. These findings support the role of explainable artificial intelligence as a decision-support tool in hematopathology rather than a standalone diagnostic system.

## Highlights

• Interpretable deep learning classified 21 bone marrow cell types across 171,373 annotated images.
• EfficientNet-B3 was benchmarked against ResNet50 and DenseNet121 using cross-validation.
• Grad-CAM, SHAP, PCA, UMAP, and t-SNE characterized model behavior and feature attribution.
• Errors clustered among morphologically adjacent cell types, reflecting cytomorphologic overlap.
• The framework supports transparent AI-assisted bone marrow cytomorphology classification.

## Introduction

Microscopic examination of bone marrow aspirates remains a cornerstone in the diagnosis and monitoring of hematological disorders, including acute leukemias, myelodysplastic syndromes, and reactive marrow conditions. Accurate identification of hematopoietic cell types relies on nuanced assessment of nuclear morphology, chromatin texture, cytoplasmic granularity, and maturation stage. However, this process is inherently subjective and is challenged by substantial morphological overlap between adjacent developmental stages, particularly within granulocytic and erythroid lineages. Interobserver variability in cytomorphological interpretation has been documented, especially in borderline or transitional forms, underscoring the need for computational tools that can assist with reproducible classification and educational reinforcement.^1^, ^2^

Recent advances in deep learning have demonstrated strong potential for automated hematopoietic cell classification from bone marrow smear images. Convolutional neural networks (CNNs) trained on large annotated datasets have achieved high overall classification performance, establishing feasibility for automated cytomorphological recognition.^3^, ^4^, ^5^, ^6^, ^7^ However, most prior studies on CNN-based hematopoietic or bone marrow cell classification have emphasized aggregate performance metrics such as accuracy, precision, recall, and F1 score, often without systematic evaluation of the learned feature space or detailed interpretability analyses. For example, in large-scale CNN classifiers for bone marrow cell morphology and related blood smear tasks.^7^, ^8^, ^9^ In digital pathology, particularly in hematopathology where subtle morphological transitions are clinically meaningful, understanding why a model classifies a cell in a certain way is as important as the classification itself.^10^, ^11^, ^12^, ^13^

Interpretability techniques such as Gradient-weighted Class Activation Mapping (Grad-CAM) and SHapley Additive exPlanations (SHAP) have increasingly been applied in histopathology to localize salient regions and quantify feature contributions.^14^, ^15^, ^16^ Nevertheless, these methods are frequently presented as isolated qualitative examples rather than integrated components of a structured analytic framework. Moreover, few studies have systematically examined how deep learning models organize hematopoietic cell types in latent embedding space using dimensionality reduction techniques such as principal component analysis (PCA), t-distributed stochastic neighbor embedding (t-SNE), or Uniform Manifold Approximation and Projection (UMAP). Latent feature geometry may provide insights into biological maturation continua, morphological adjacency, and systematic sources of misclassification.^17^, ^18^, ^19^

In addition, many published cytomorphology classification studies rely on single train–validation splits without cross-validation, limiting assessment of model stability and generalizability. Given known class imbalance in bone marrow datasets where granulocytic and erythroid precursors may predominate robust evaluation using macro-averaged metrics and cross-validation is necessary to ensure equitable assessment across all cell types.^20^, ^21^, ^22^

Our study reframes multiclass bone marrow cytomorphology classification as an interpretability-centered investigation rather than a purely performance-driven comparison. Using a dataset of 171,373 annotated bone marrow cell images spanning 21 hematopoietic classes, we benchmark EfficientNet-B3 against ResNet50 and DenseNet121 architectures. We integrate: (1) full per-class evaluation metrics, (2) 5-fold stratified cross-validation to assess performance stability, (3) latent embedding visualization via PCA, t-SNE, and UMAP, and (4) structured post hoc interpretability analysis using Grad-CAM and SHAP focused on high-frequency misclassification pairs.

Our objective is to provide a systematic framework for evaluating latent feature organization, confusion structure, and attribution behavior in deep learning models applied to hematopathology. By emphasizing interpretability, embedding geometry, and model stability, this study aims to contribute methodological clarity to the development of clinically meaningful computational tools in bone marrow cytomorphology.

## Methods

### Dataset and cohort definition

We utilized the publicly available Bone Marrow Cytomorphology dataset developed by Matek et al., consisting of 171,373 single-cell images across 21 morphological classes derived from digitized peripheral blood and bone marrow smears. Images were presegmented at the single-cell level and annotated by expert hematopathologists.^23^ The dataset was split into training and validation partitions using a fixed seed (42) with approximately 80% for training (*n* = 137,099) and 20% for validation (*n* = 34,274). All models were evaluated on the identical held-out validation set to enable paired statistical comparison. Class distribution was moderately imbalanced, with granulocytic and erythroid precursors comprising the largest categories. No external dataset was available for independent validation; therefore, robustness was assessed via stratified k-fold cross-validation.

### Image preprocessing and data augmentation

All images were resized to 300 × 300 pixels and normalized to ImageNet statistics. During training, we applied random horizontal and vertical flips, random rotations, random brightness and contrast jitters, random cropping with scaling augmentations. Augmentation was applied to mitigate overfitting and improve robustness to smear preparation variability. No augmentation was applied during validation.

### Model architecture and training

To evaluate architectural performance differences, three CNNs were implemented, EfficientNet-B3, ResNet50, and DenseNet121.^24^, ^25^, ^26^ All models were initialized with ImageNet pretrained weights and modified with a final fully connected layer containing 21 output neurons corresponding to the morphological classes. EfficientNet-B3 was selected as the primary architecture due to its compound scaling strategy, which balances depth, width, and resolution and has demonstrated strong parameter efficiency across vision tasks.^24^ ResNet50 and DenseNet121 were included as comparator architectures due to their widespread use in digital pathology literature. Dropout of 0.3 was applied in the classification head to reduce overfitting.

Models were trained using Adam optimizer, initial learning rate of 1e-3, fine-tuning phase learning rate - 1e-5 batch size = 32, cross-entropy loss. Early stopping was applied based on validation loss. Class weights were evaluated in secondary experiments; however, baseline results are reported for comparability across architectures.

### Cross-validation and performance metrics

To assess robustness, 5-fold stratified cross-validation was performed on a balanced subset (*n* = 34,626; approximately 2380 images per class). Each fold was trained for five epochs. Mean and standard deviation of top-1 accuracy, macro-F1, and weighted-F1 were computed. Cross-validation reduces variance in performance estimates and mitigates partition bias. Performance was evaluated using top-1 accuracy, top-5 accuracy, macro-average F1 score, weighted F1 score, per-class F1 scores, and confusion matrices of all 21 classes were evaluated equally.

### Statistical comparison between architectures

Formal paired statistical comparison was performed on the held-out validation set. McNemar's exact test was used to compare paired prediction outcomes between models, evaluating discordant prediction pairs (n01,n10). This test determines whether two classifiers differ significantly on identical samples. Bootstrap resampling (2000 iterations) was used to compute 95% confidence intervals for differences in accuracy, macro-F1 and weighted-F1. Bootstrapping was performed with stratified sampling to preserve class proportions. This approach provides a non-parametric estimate of performance difference uncertainty.

### Model interpretability

Grad-CAM^14^ was applied to visualize spatial regions contributing to classification decisions. Grad-CAM maps were generated for top five most frequent confusion pairs and representative correct predictions. Sixty high-confidence misclassified samples per confusion pair were screened, from which 25 representative images were selected for figure generation. Grad-CAM visualizations were generated for 25 representative images selected from the most frequent confusion pairs and correctly classified examples. Grad-CAM heatmaps were overlaid on original images and stitched into composite panels. SHAP analysis was performed on the 1536-dimensional latent feature embeddings extracted from the penultimate EfficientNet-B3 layer. A surrogate logistic regression model was trained on these embeddings and used to estimate SHAP values, quantifying the contribution of latent embedding dimensions to classification predictions rather than individual image pixels.^15^ Global feature importance was summarized using mean absolute SHAP values across the evaluation subset. SHAP analysis was performed on a representative subset of 63 validation samples comprising high-confidence correct classifications, high-confidence misclassifications, and dominant confusion pairs. Due to the computational cost of SHAP analysis on high-dimensional embeddings, this subset was selected using stratified sampling across classes.

### Embedding analysis and analytic tools

To examine feature space separability, embeddings from the penultimate layer were projected into two dimensions using, PCA, UMAP, and t-SNE. Projection plots were generated for each architecture and compared qualitatively. All experiments were implemented in Python using, PyTorch, timm, scikit-learn, umap-learn, and SHAP. Random seeds were fixed (42) for reproducibility. Configuration files, train-validation manifests, and supplementary analysis outputs are provided in the supplementary materials.

## Results

### Dataset characteristics

The dataset comprised 171,373 single-cell images across 21 hematopoietic morphological classes. The validation set contained 34,274 images. Class distribution was imbalanced, with granulocytic and erythroid precursors comprising the largest proportions. Supplementary Figs. S1–S5 provide additional Grad-CAM visualizations across EfficientNet-B3, ResNet50, and DenseNet121 architectures. This imbalance was consistent across train and validation splits due to stratified partitioning.

### Model performance

#### EfficientNet-B3 (primary model)

**Fig. 1:**
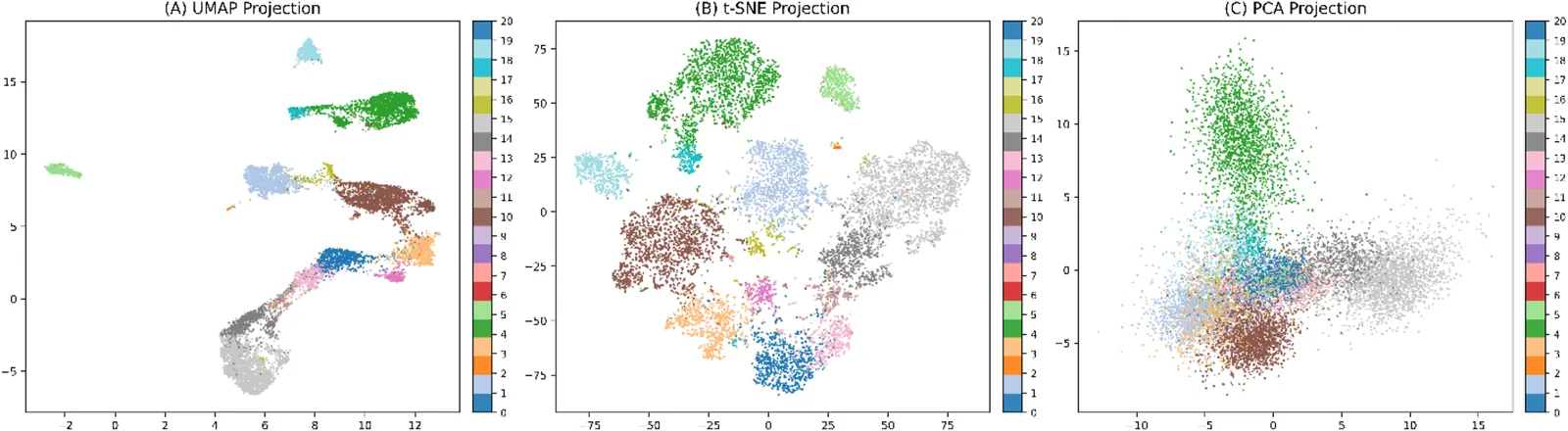
Latent embedding visualization of bone marrow cytomorphology representations learned by EfficientNet-B3. Penultimate-layer embeddings from the trained EfficientNet-B3 model were projected into two dimensions using three dimensionality-reduction techniques: principal component analysis (PCA), Uniform Manifold Approximation and Projection (UMAP), and t-distributed stochastic neighbor embedding (t-SNE). Each point represents a single cell image colored by ground-truth morphological class. Distinct clustering is observed for several mature hematopoietic populations, whereas transitional developmental stages exhibit partial overlap, reflecting the known biological continuum of hematopoietic maturation.

**Fig. 2:**
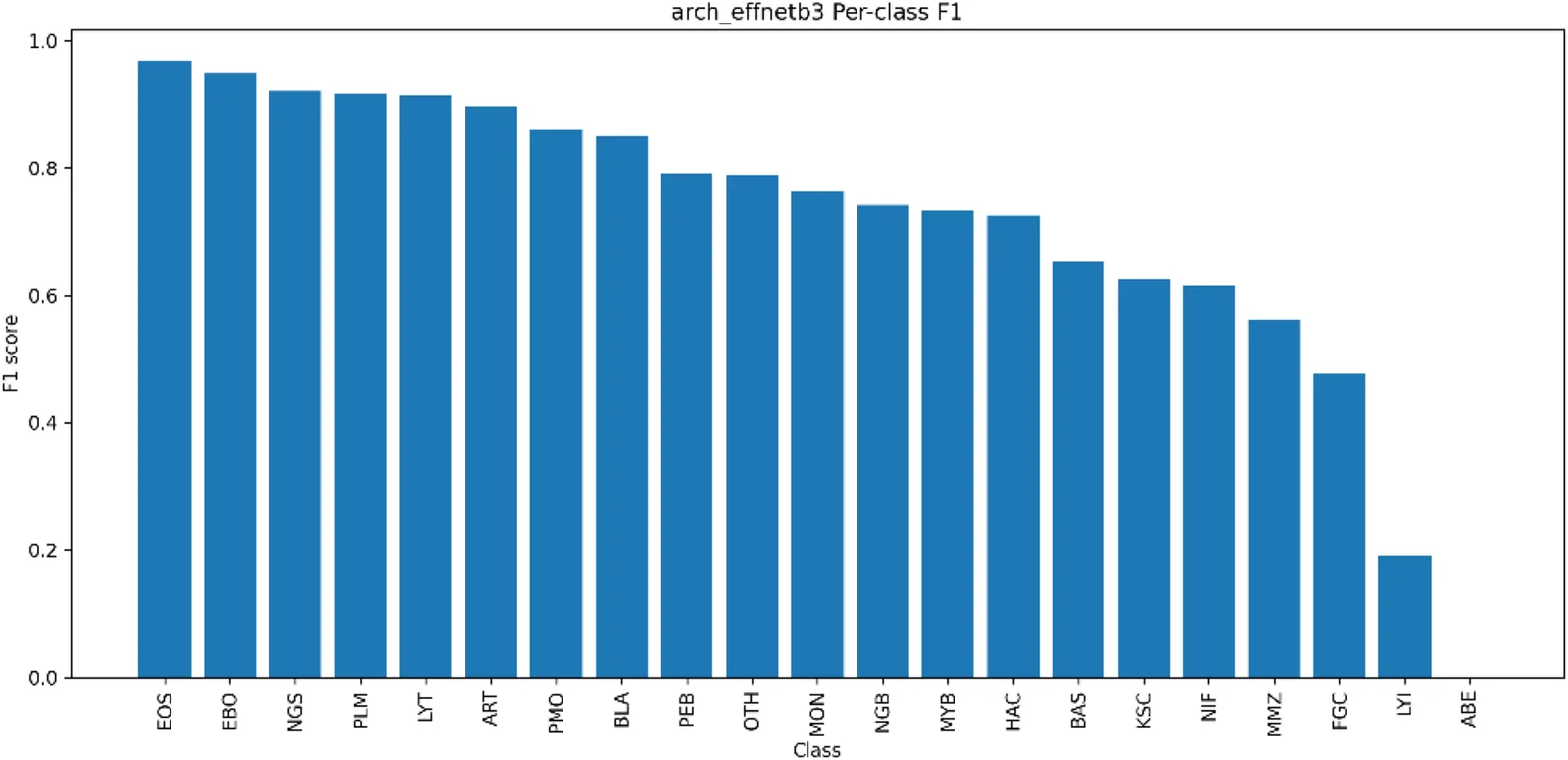
Per-class F1 score distribution for EfficientNet-B3 across 21 hematopoietic morphological classes. Class-specific F1 scores derived from the held-out validation set illustrate variation in classification performance across morphological categories. Mature granulocytic and erythroid lineages demonstrate higher F1 scores, whereas transitional precursor classes show reduced performance due to morphological similarity between adjacent maturation stages.

On the held-out validation set, EfficientNet-B3 achieved: top-1 accuracy: 87.6%, top-5 accuracy: 99.4%, macro-F1 score: 0.712, weighted-F1 score: 0.875 as shown in Table 1. Whereas top-5 accuracy approached 99%, top-1 accuracy reflects more clinically relevant single-label performance and is therefore emphasized. Per-class F1 analysis demonstrated strong performance for granulocytic and erythroid lineages but reduced performance in morphologically overlapping categories such as segmented neutrophil (NGS) ↔ neutrophilic band (NGB), MYB ↔ PMO and EBO ↔ PEB. Fig. 1 shows PCA, UMAP, and t-SNE projections of EfficientNet-B3 penultimate layer embeddings. Full confusion matrices for all three architectures are provided in Supplementary Figs. S8–S10. These confusion pairs correspond to known morphological similarity in early hematopoietic differentiation. Fig. 2 shows per-class F1 performance for EfficientNet-B3.

**Table 1:** Comparative classification performance of EfficientNet-B3, DenseNet121, and ResNet50 on the held-out validation set.

Model	Top-1	Macro-F1	Weighted-F1
EfficientNet-B3	87.6%	0.712	0.875
DenseNet121	87.4%	0.721	0.873
ResNet50	87.0%	0.718	0.868

### Statistical comparison of CNN architectures

Pairwise statistical comparisons were performed using aligned prediction outputs from the three CNN architectures on a paired evaluation set. EfficientNet-B3 demonstrated statistically significant improvements relative to both ResNet50 and DenseNet121 according to McNemar's test and paired bootstrap analyses. Detailed statistical comparison results are summarized in Supplementary Table S5, with complete paired comparison outputs provided in Supplementary Files 5 and 6. Because the paired statistical comparison outputs were generated from a separate aligned prediction export (*n* = 34,275 paired samples), these analyses are presented as supplementary sensitivity analyses and are not used to replace the primary held-out validation performance metrics reported above.

### Comparative architecture performance

Performance comparison across architectures is summarized in Table 1.

EfficientNet-B3 achieved the highest top-1 and weighted-F1 scores. DenseNet121 demonstrated slightly higher macro-F1, suggesting improved minority-class balance. Confusion matrices for all three architectures are shown in Supplementary Fig. S8–10.

### Cross-validation analysis

5-fold stratified cross-validation was performed on a balanced subset (*n* = 34,626). Mean performance across folds:

**Table:** 

Model	Top-1 (mean ± SD)	Macro-F1 (mean ± SD)
EfficientNet-B3	0.763 ± 0.003	0.663 ± 0.007
ResNet50	0.765 ± 0.004	0.625 ± 0.010
DenseNet121	0.776 ± 0.005	0.682 ± 0.011

DenseNet121 demonstrated slightly higher cross-validated macro-F1, suggesting improved generalization on balanced subsets (Supplementary Table S1). Variance across folds was low (SD < 0.012), indicating stable performance.

## Interpretability results

### Embedding space analysis

To examine feature separability, penultimate-layer embeddings were projected into 2D using PCA, UMAP, and t-SNE. Fig. 1 shows EfficientNet-B3 Embedding Structure with UMAP projection, t-SNE projection, and PCA projection. Distinct clustering was observed for mature granulocytic and erythroid classes, whereas transitional classes (e.g., MYB, PMO, and MMZ) demonstrated partial overlap. This clustering pattern mirrors the observed confusion matrix structure, indicating that classification errors are associated with intrinsic morphological continuum rather than random misclassification. Dimensionality-reduction projections of EfficientNet-B3 embeddings revealed structured organization of hematopoietic cell representations (Fig. 1). Mature granulocytic and erythroid populations formed relatively distinct clusters, whereas transitional precursor stages showed partial overlap. This pattern is consistent with the biological continuum of hematopoietic maturation, where intermediate developmental stages share morphological features.

### Cross-architecture UMAP comparison

Embedding projections for EfficientNet-B3 are shown in Fig. 1, whereas corresponding projections for ResNet50 and DenseNet121 are provided in Supplementary Figs. S6 and S7. All models demonstrate similar global topology. However, EfficientNet-B3 exhibits slightly tighter cluster compactness for erythroid precursors. This suggests that architecture selection modestly influences embedding geometry but does not fundamentally alter morphological separability.

### Confusion pattern analysis

**Fig. 3:**
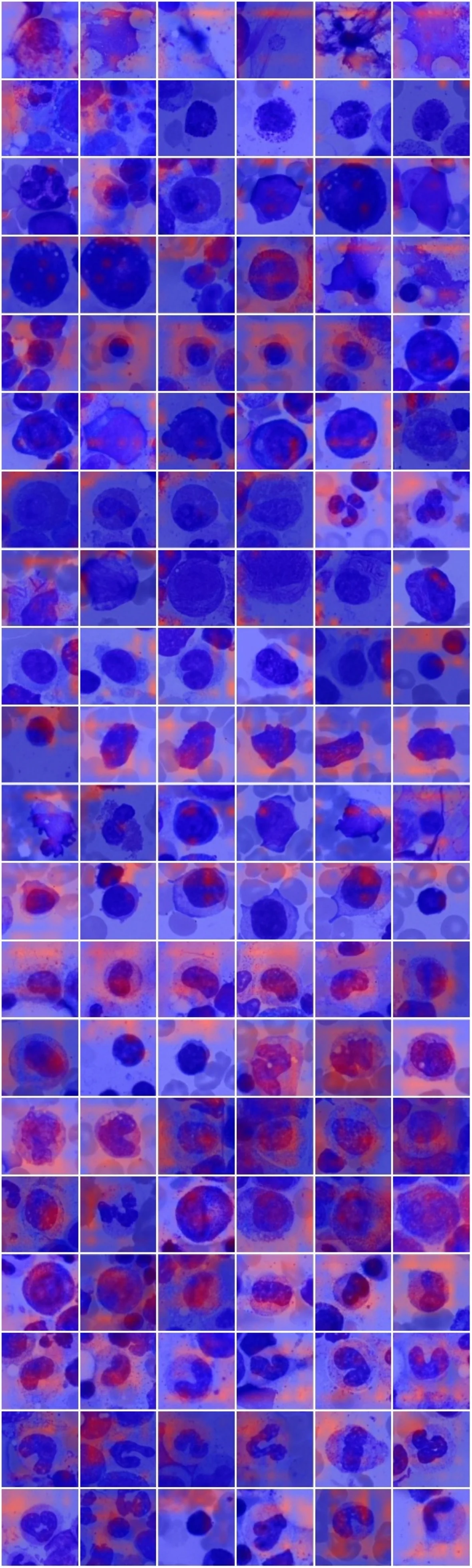
Grad-CAM visualization of EfficientNet-B3 attention regions in bone marrow cytomorphology classification. Gradient-weighted Class Activation Mapping (Grad-CAM) overlays highlight spatial regions contributing most strongly to model predictions. Attention maps consistently localize to biologically relevant morphological features including nuclear contour, chromatin texture, and cytoplasmic granularity. Examples include both correct predictions and high-confidence misclassifications from the most frequent confusion pairs.

The five most frequent confusion pairs for EfficientNet-B3 were: NGS → NGB (214 cases), NGB → NGS (208 cases), MYB → PMO (187 cases), EBO → PEB (87 cases), NGB → MMZ (85 cases). These patterns were consistent across architectures (Supplementary Tables S2–S4). Confusion panels generated via Grad-CAM are shown in Fig. 3.

### Grad-CAM interpretability

Grad-CAM overlays were generated for: 60 high-confidence misclassifications per top confusion pair and representative correct predictions. Grad-CAM visualizations demonstrated that model attention frequently localized to morphological regions consistent with diagnostically relevant features, including nuclear contours, chromatin patterns, and cytoplasmic granularity (Fig. 3). Whereas these visualizations do not provide direct mechanistic explanations, they suggest that the model focuses on spatial regions commonly used by hematopathologists during morphological assessment. Grad-CAM localization was qualitatively consistent across architectures. Additional Grad-CAM examples are provided in Supplementary Fig. S2.

### SHAP feature attribution

**Fig. 4:**
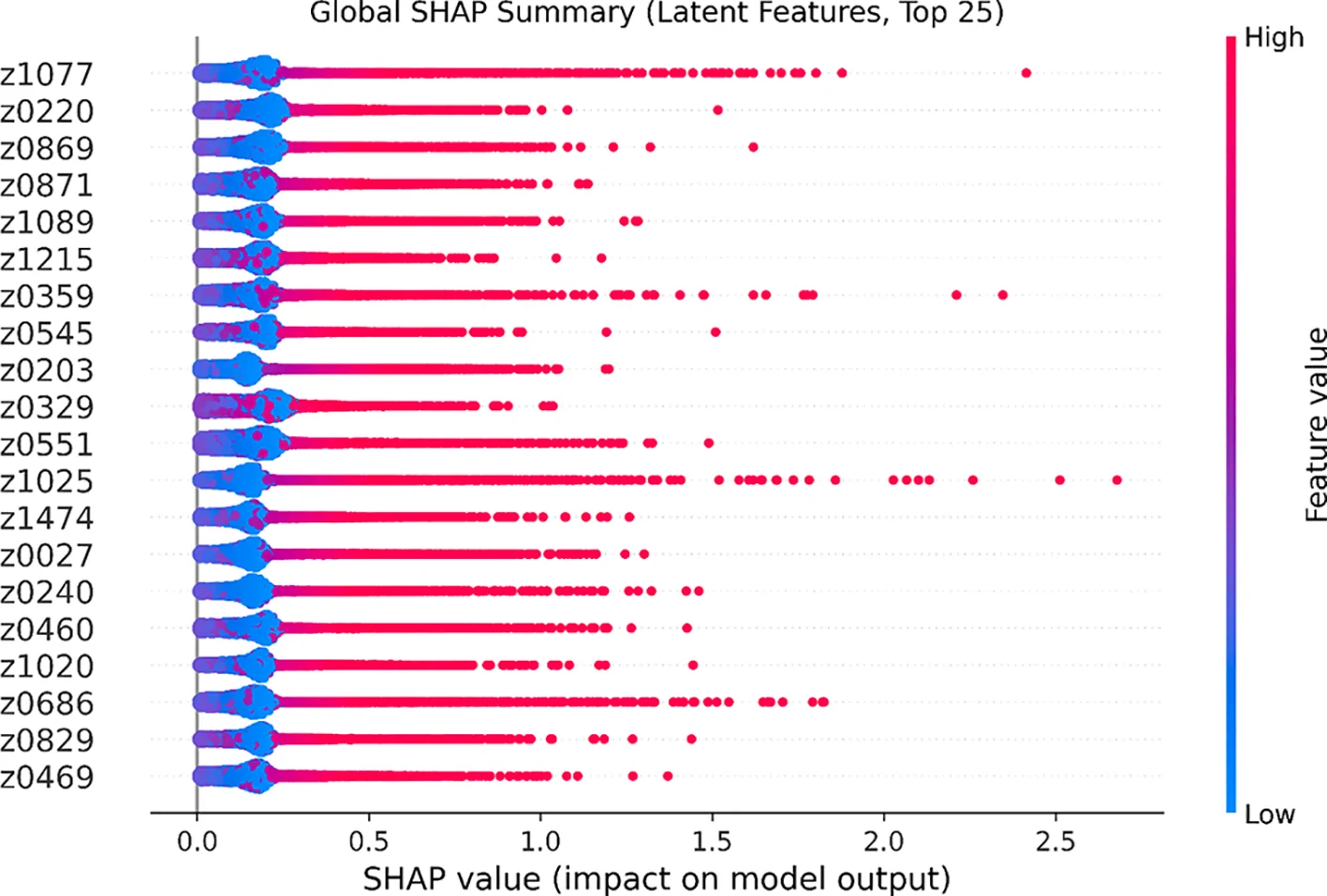
Global SHAP feature importance for EfficientNet-B3 latent embeddings. Mean absolute SHAP values are shown for the 25 most influential embedding dimensions. Features labeled *z####* correspond to coordinates of the 1536-dimensional latent representation extracted from the EfficientNet-B3 penultimate layer. Higher absolute SHAP values indicate latent embedding dimensions that exert greater influence on classification predictions across the evaluation dataset.

SHAP analysis was performed on 63 representative validation samples. Global SHAP analysis of EfficientNet-B3 embeddings revealed that a limited subset of latent dimensions contributed disproportionately to classification decisions (Fig. 4). Feature contribution distributions were similar across architectures, supporting architectural consistency in learned morphological representations.

Across analyses, a consistent pattern emerged linking model performance, embedding structure, and interpretability results. Classes that formed well-separated clusters in embedding projections generally exhibited higher per-class F1 scores and fewer confusion matrix errors, whereas morphologically transitional stages showed both embedding overlap and increased misclassification frequency. Grad-CAM visualizations for these confusion pairs frequently highlighted subtle nuclear segmentation differences and chromatin patterns, reflecting the morphological continuum between adjacent developmental stages. Global SHAP analysis further demonstrated that a limited subset of latent embedding dimensions contributed disproportionately to classification predictions. Taken together, these findings indicate that model errors are largely structured along biologically meaningful hematopoietic maturation trajectories rather than arising from random classifier instability.

### Clinical interpretation of performance

Although top-5 accuracy exceeded 99%, top-1 accuracy (∼87%) indicates that the model remains insufficient as a standalone clinical diagnostic tool. However, error patterns were concentrated in biologically adjacent developmental stages, suggesting that misclassifications largely occur along expected hematopoietic differentiation trajectories rather than across unrelated lineages. This reinforces the value of interpretability-focused analysis in understanding model behavior within morphological continua.

## Discussion

In this study, we developed and evaluated an interpretable deep learning framework for multiclass hematopoietic cell classification using EfficientNet-B3 and complementary post hoc interpretability methods. Across 21 morphological categories, EfficientNet-B3 achieved a top-1 accuracy of 87.6% and top-5 accuracy of 99.4%, with consistent performance across stratified cross-validation. Comparative evaluation demonstrated consistent performance advantages relative to ResNet50 and DenseNet121 across supplementary paired analyses. These findings support the selection of EfficientNet-B3 as the primary architecture for interpretability-focused evaluation while acknowledging that the primary performance estimates are derived from the held-out validation cohort.

More importantly, embedding visualization, confusion pattern analysis, Grad-CAM attention mapping, and SHAP feature attribution collectively demonstrate that classification errors are largely confined to morphologically adjacent developmental stages rather than unrelated lineages. This suggests that misclassification reflects biological continuum rather than model instability.

### Hematopoietic morphology as a challenging domain

Hematopoietic cell classification is uniquely challenging compared to other pathology tasks due to: continuous morphological differentiation along lineage trajectories, subtle nuclear chromatin changes across maturation stages, cytoplasmic granularity overlap between transitional forms and inherent interobserver variability in manual classification. Previous studies have reported similar difficulty distinguishing early granulocytic and erythroid precursors.^4^, ^6^ In particular, granulocytic stages such as NGB and NGS exhibit minimal morphological separation, which is reflected in our dominant confusion pairs.

Matek et al.^8^, ^23^ demonstrated high performance in bone marrow classification using deep learning but also noted performance degradation among transitional cell types. Our findings align with these observations and extend them through systematic interpretability analysis. The observed embedding overlap in UMAP and t-SNE projections reinforces that classification boundaries reflect morphological continuum rather than discrete clusters.

### Comparison with prior deep learning approaches

EfficientNet architectures have demonstrated strong performance across medical imaging tasks due to compound scaling strategies that balance depth, width, and resolution.^24^ However, prior hematopathology and leukocyte-classification studies have predominantly adopted ResNet or DenseNet backbones rather than EfficientNet, as seen in white blood cell classifiers built on pretrained ResNet and DenseNet, leukocyte CAD systems based on DenseNet-161, and systematic benchmarks in which DenseNet-169 and ResNet-101 serve as primary architectures.^27^, ^28^, ^29^ In this study, EfficientNet-B3 achieved marginally higher top-1 accuracy than both ResNet50 and DenseNet121, although the magnitude of improvement over DenseNet121 was modest. These findings suggest that whereas EfficientNet provides incremental improvements, architecture choice alone does not fundamentally resolve morphological ambiguity. This reinforces the need to shift focus from purely optimizing accuracy to understanding model decision processes.

### Interpretability as a central contribution

Interpretability remains a critical barrier to clinical adoption of artificial intelligence (AI) in pathology.^10^, ^30^, ^31^ Grad-CAM visualizations in this study demonstrate that the model predominantly attends to biologically meaningful structures such as nuclear contour irregularities, chromatin condensation, cytoplasmic granularity. Importantly, misclassified NGS ↔ NGB samples often showed attention concentrated on ambiguous nuclear segmentation, mirroring known diagnostic challenges. A small subset of embedding dimensions demonstrated consistently high mean absolute SHAP values, indicating that classification decisions relied on a limited number of latent morphological representations. By combining global embedding projections (PCA/UMAP/t-SNE) with local interpretability (Grad-CAM and SHAP), this framework provides a multiscale explanation of model behavior, an approach rarely integrated systematically in hematopathology studies. Integrating latent embedding analysis, Grad-CAM visualization, and SHAP-based feature attribution provides a complementary multilevel interpretability framework for deep learning models applied to bone marrow cytomorphology.

### Clinical relevance and limitations of accuracy

Whereas top-5 accuracy exceeded 99%, top-1 accuracy (∼87%) remains insufficient for standalone clinical deployment. However, this performance must be contextualized. First, interobserver agreement among hematopathologists for transitional forms is not perfect.^32^, ^33^ Many “errors” may reflect inherent ambiguity rather than true misclassification. Similar to prior work in blood-cell image analysis where misclassifications cluster among developmentally related, morphologically similar granulocytic stages, our model's errors are concentrated within biologically adjacent classes, suggesting that it preserves lineage topology rather than producing random misassignments.^34^ Consistent with current perspectives on AI in medical imaging and hematology, such systems are better positioned as decision-support tools that augment, rather than replace, expert review.^35^ Nevertheless, rigorous clinical validation on independent, multi-institutional cohorts remains essential before translational deployment, as emphasized in recent guidance on accountable AI in medical imaging and deep learning in hematology.^35^

### Dataset imbalance and generalization

Class imbalance is intrinsic to hematopoietic populations, where common lineages dominate bone marrow smears and certain diagnostically important subsets are rare.^35^ Although stratified sampling and weighted metrics were used, macro-F1 scores remained lower than weighted-F1 scores, consistent with prior observations that performance varies substantially across bone marrow cell types in imbalanced datasets.^21^, ^36^ Cross-validation demonstrated stable variance (SD < 0.012), suggesting robustness across folds. Future work could therefore investigate class-balanced and focal loss formulations, which have improved performance on class-imbalanced medical imaging benchmarks. Synthetic augmentation for rare cell types for example using GAN-generated bone marrow smear images to bolster underrepresented classes has shown promise in improving classifier training.^37^, ^38^

### Limitations and future work

First, the model was trained and evaluated on a single institutional dataset, and no external validation cohort was available. As a result, generalizability to other labs, scanners, and staining protocols remains uncertain, and future work should include fully segregated external test datasets to substantiate performance claims. Although cross-validation was implemented, a fully segregated external dataset would strengthen generalizability claims.^39^, ^40^

Our model uses morphological image data alone and does not integrate clinical variables such as complete blood counts, flow cytometry, or molecular genetic information, which are routinely used by hematopathologists in practice. Multimodal models that combine morphology with relevant clinical context may further improve diagnostic performance.^35^ Moreover, our interpretability analyses rely on post hoc methods (Grad-CAM and SHAP). Whereas these techniques highlight salient regions and feature contributions, they do not provide causal explanations and may not always faithfully reflect the model's internal reasoning, as emphasized in critiques of post hoc explainability for high-stakes decision making.^31^ Despite these limitations, our study advances beyond prior classification-only work by embedding performance evaluation within a comprehensive interpretability framework, including systematic visualization of saliency maps, feature attributions, and latent embedding structure. This combined analysis offers more granular insight into model behavior than aggregate accuracy metrics alone.^35^

Future work should focus on multicenter validation, multimodal integration (clinical + morphological features), self-supervised pretraining strategies, trajectory-aware classification that models hematopoietic lineage continuity. Additionally, embedding topology could be used to quantify morphological progression along differentiation pathways, bridging machine learning with hematological biology.

## Conclusion

This study demonstrates that, in the context of morphologically continuous hematopoietic cell populations, incremental architectural refinements yield only modest performance gains. Whereas interpretability analyses provide substantially deeper insight into model behavior. By integrating statistical comparison with latent embedding visualization and multilevel interpretability analysis, we propose a framework that explicitly prioritizes transparency and biological plausibility over raw accuracy alone. Such approaches are likely to be essential for the development of trustworthy AI systems in hematopathology, where subtle morphological transitions and clinically meaningful edge cases demand models whose decision processes can be interrogated and aligned with domain knowledge.

## Ethics, consent to participate

This study was conducted using publicly available, fully de-identified data and did not require IRB review under local regulations.

## Consent to publish declarations

All authors reviewed the manuscript and provided their consent for publication.

## CRediT authorship contribution statement

Deidentified public-use secondary data were analyzed, and therefore no protocol approval was required.

Sulley, Saanie Conceptualization, Data curation, Formal analysis, Investigation, Methodology, Visualization, Writing - original draft, Writing - review & editing. Ghassan Tranesh - Conceptualization, Writing - review & editing.

## Clinical trial number

Not applicable.

## Funding

This research did not receive any specific grant from funding agencies in the public, commercial, or not-for-profit sectors.

## Declaration of competing interest

The authors declare that they have no known competing financial interests or personal relationships that could have appeared to influence the work reported in this article.

## Data Availability

The publicly available data files for the Bone-Marrow Cytomorphology_MLL_Helmholtz_Fraunhofer: An Expert-Annotated Dataset of Bone Marrow Cytology in Hematologic Malignancies from the Cancer Imaging Archive were used. The data can be obtained through the Cancer Imaging Archive website. The datasets are available through the link here, https://doi.org/10.7937/TCIA.AXH3-T579. Code for analysis and visualization is available upon reasonable request.

## Appendix A Supplementary data

Model performance comparison across EfficientNet-B3, DenseNet121, and ResNet50 architectures and secondary augmentation/class-weighting experiments.

Most frequent class-level misclassification pairs for EfficientNet-B3 on the held-out validation set.

Most frequent class-level misclassification pairs for ResNet50 on the held-out validation set.

Most frequent class-level misclassification pairs for DenseNet121 on the held-out validation set.

Paired statistical comparison of EfficientNet-B3 and ResNet50 using aligned prediction outputs, McNemar testing, and paired bootstrap confidence intervals.

Paired statistical comparison of EfficientNet-B3 and DenseNet121 using aligned prediction outputs, McNemar testing, and paired bootstrap confidence intervals.

Fixed-seed (seed 42) train-validation split manifest for the 171,373 bone marrow cytomorphology images used in the study.

Training and analysis configuration file for the EfficientNet-B3 architecture, including preprocessing and reproducibility parameters.

Training and analysis configuration file for the ResNet50 architecture, including preprocessing and reproducibility parameters.

Training and analysis configuration file for the DenseNet121 architecture, including preprocessing and reproducibility parameters.

Supplementary Table S5. Paired statistical comparison of CNN architectures using aligned prediction outputs
