# Supplementary material for: An interpretable deep learning framework for multiclass bone marrow cytomorphology classification using EfficientNet and post hoc visualization techniques
Source: J Pathol Inform. 2026 Aug 20;23:100710. doi: 10.1016/j.jpi.2026.100710 (PMC13578374; doi:10.1016/j.jpi.2026.100710)
Supplement: Supplementary file 1 [file mmc11.docx]

| **Comparison** | **Accuracy Δ** | **Weighted F1 Δ** | **McNemar p-value** | **95% CI** |
| --- | --- | --- | --- | --- |
| EffNet-B3 vs ResNet50 | +1.37% | +1.49% | <0.001 | 1.11%-1.65% |
| EffNet-B3 vs DenseNet121 | +0.62% | +0.63% | <0.001 | 0.36%-0.88% |

Supplementary Table S5. Paired statistical comparison of CNN architectures using aligned prediction outputs. McNemar’s test and paired bootstrap analyses were performed on a paired evaluation set (n = 34,275) to compare EfficientNet-B3 against ResNet50 and DenseNet121. Primary model performance metrics are reported separately from the held-out validation cohort.
